# Complete Genome Sequence of Ralstonia pseudosolanacearum Strain Sw698, Isolated from Solanum lycopersicum

**DOI:** 10.1128/mra.00942-22

**Published:** 2023-04-27

**Authors:** HwangWeon Jeong, Jae-Hyeon Oh, Sung-Mi Kim, Young-Joo Seol, Hye-Jin Yoon, Jinjeong Lee, Su Jung Ra, Eunhee Kim, Myeong Jin Kang, Il-Pyung Ahn

**Affiliations:** a National Institute of Agricultural Sciences, Jeonju, South Korea; University of Arizona

## Abstract

Ralstonia pseudosolanacearum is a member of the Ralstonia solanacearum species complex (RSSC), which is composed of three species and diverse subspecific groups. Some strains cause bacterial wilt in Solanum lycopersicum; others are beneficial for their hosts. Herein, we present the complete genome sequence of an RSSC strain, Sw698, beneficial for *S. lycopersicum* growth.

## ANNOUNCEMENT

Ralstonia pseudosolanacearum belongs to the Ralstonia solanacearum species complex (RSSC) (1). Although the RSSC comprises notorious bacterial wilt pathogens infecting more than 200 plant species (2, 3), considerable proportions of the RSSC also reside *in planta* or around the rhizosphere of host plants without causing any observable alterations (4). This mode is a typical example of commensalism. Further, some strains prefer mutualism to parasitism in the relationship with their hosts and enhance plant growth via shaping host-beneficial conditions. The widely diverse interaction modes of RSSC strains with their hosts, ranging from parasitism to mutualism, suggest a wide spectrum of subspecific RSSC divergence under given hosts and microbial environments (5). Some RSSC strains have evolved typical parasitism-specific pathogenicity systems, and others have developed a symbiotic apparatus. Our strain, Sw698, was isolated from the vascular tissue of tomato plants exhibiting bacterial wilt symptoms (6); however, this strain evidently enhanced the growth of Solanum lycopersicum. Therefore, the microbiome of the symptomatic tissue could comprise both symbiotic RSSC strains and parasitic ones.

*R. pseudosolanacearum* strain Sw698, retrieved from the vascular tissues of diseased tomato plants in South Korea, was obtained from the Korean Agricultural Culture Collection (KACC; number 10698). A single colony of Sw698 was streaked onto casamino acid-peptone-glucose agar (CPGA) (7) and grown at 28°C for 48 h. The propagated cells were harvested from the CPGA for DNA preparation (Nanobind CBB kit; Pacific Biosciences, CA, USA). After confirmation of the DNA quality (Femto Pulse system; Agilent, CA, USA), a Sequel library was constructed following the 15-kbp template library preparation workflow (SMRTbell prep kit 3.0; Pacific Biosciences, CA, USA) and sequenced on the Sequel sequencing platform (Pacific Biosciences, CA, USA). Sequencing yielded 168,544 analyzed subreads and 2,406,191,173 bases (*N*_50_ = 15,583 bp). *De novo* assembly through the Microbial Assembly application, a part of the PacBio_SMRT program, produced two circular contigs. A TruSeq library was also prepared from the same DNA (TruSeq Nano DNA high-throughput library prep kit; Illumina, CA, USA) and sequenced on a HiSeq X instrument (Illumina). The analysis resulted in a total of 8,260,550 reads and 1,245,407,029 bases, with a Q30 value of 97.4%. The TruSeq reads were applied to the Sequel contigs using Pilon v1.21 with default parameters for assembly validation.

The two Sequel contigs, a chromosome and a megaplasmid, comprised 3,450,728 bp and 2,111,456 bp, with GC contents of 67.0% and 67.1%, respectively. Their reading depths were 170.9× and 306.6×. Annotation based on the Prokaryotic Genome Annotation Pipeline (8) predicted 3,184 coding DNA sequences (CDSs), 59 tRNAs, and 9 rRNAs on the chromosome and 1,675 CDSs, 7 tRNAs, and 3 rRNAs on the megaplasmid. To unravel the reasons behind the avirulence of Sw698 on tomatoes, the genes involved in the protein secretion system and encoding type 3 effectors were identified using TXSScan (9) and Ralsto T3E (10). Sw698 harbors 161 genes of the type 1 secretion system (T1SS; 29 genes), T2SS (27 genes), T3SS (15 genes), T4SS (28 genes), T5SS (12 genes), T6SS (16 genes), and flagella/pilli (17/17 genes). The virulent type strain GMI1000 contains 192 genes encoding these systems. Whereas 71 genes encode type 3 effectors in GMI1000, Sw698 comprises only 50 of these genes.

### Data availability.

The genome information for the Sw698 chromosome and megaplasmid was deposited at GenBank under accession numbers CP104498 and CP104499. The TruSeq and Sequel raw sequence reads were submitted to the NCBI under SRA accession numbers SRR20083558 to SRR20083561, BioProject accession number PRJNA857834, and BioSample accession number SAMN29638280.
